# Rapid Shallow Breathing Index as a Predictor of Extubation Outcomes in Pediatric Patients Underwent Cardiac Surgeries at King Faisal Cardiac Center

**DOI:** 10.7759/cureus.8754

**Published:** 2020-06-21

**Authors:** Farid A Munshi, Ziad M Bukhari, Hassan Alshaikh, Majd Saem Aldahar, Turki Alsafrani, Mostafa Elbehery

**Affiliations:** 1 Pediatric Cardiac Critical Care Unit, King Faisal Cardiac Center, King Abdulaziz Medical City, Jeddah, SAU; 2 Medicine, King Saud Bin Abdulaziz University for Health Sciences, Jeddah, SAU

**Keywords:** rsbi, cardiac surgery, extubation failure, pediatric

## Abstract

Introduction

Weaning patients of ventilation is an important step in the intensive care unit; therefore, assessing the perfect timing to do such critical action is of equal significance to prevent complications. Rapid shallow breathing index (RSBI) has been used as a prediction tool for weaning adult patients, but for pediatric patients it is still an area of unclarity. Accordingly, the aim of this study is to evaluate the RSBI as a predictor of extubation outcome in pediatric patients underwent cardiac surgery at King Faisal Cardiac Center from 2016 until 2019.

Methods

A retrospective cohort study was conducted at King Faisal Cardiac Center on all extubated children having cardiac surgeries from 2016 to 2019 with excluding the patients who were admitted for causes other than cardiac surgery. Their age was ranged from birth until 14 years. Moreover, the patients were grouped based on the extubation outcomes into: success, success with non-invasive ventilation, or failure which was defined as reintubation within 48 hours after extubation. Regarding the collected data, three readings of RSBI on hourly basis prior to extubation were calculated by dividing respiratory rate (RR) over tidal volume (VT) with a correction based on the body weight.

Results

A total of 86 patients met the inclusion and exclusion criteria. Thirty (34.9%) patients were successfully extubated, 51 (59.3%) patients had successful extubation with the use of non-invasive ventilation, and only five (5.8%) patients suffered from extubation failure. Two-hour RSBI as a predictor of outcome had a P-value of 0.003, one-hour RSBI had a P-value of 0.01, RSBI at time of extubation had a P-value of 0.02. Mean corpuscular volume (MCV) is higher in extubation failure group with a p-value of 0.01.

Conclusion

This study suggests that pediatric patients who suffer from extubation failure usually have a higher RSBI measurement compared to the patients who have a successful extubation. The most significant RSBI measurements to predict the extubation outcome were recorded two hours prior to extubation. Our study also found that extubation failure patients could have higher MCV than the success group.

## Introduction

Out of all patients in the intensive care unit (ICU), 40% of patients may require mechanical ventilation (MV) [1]. In the last 10 years, the percentage of pediatric patients who required MV has increased by 11% [2]. While MV is a way to maintain normal breathing, prolongation may lead to multiple complications such as ventilator-induced pneumonia (10-20%), airway injuries, mucosal/dermal pressure ulcer (20%), vocal cord paralysis (0.03%), and sinusitis (0.12%) [2,3]. Late complications of prolonged MV may occur days to weeks after extubation which are obstructive fibrinous tracheal pseudo membrane, post intubation tracheal stenosis, and tracheomalacia [3]. Although they are rare yet, presence of such complications may be fatal. On the other hand, early extubation of MV may decrease the risk of getting such complications and could lead to low mortality rate (1%), short length of ICU stay (one day), and less hospital stay (four days) [4]. Among pediatric ICU patients, 10 to 20% may suffer from extubation failure, which is the need of reintubation within 48 hours after MV is removed [5,6]. For that reason, prediction of the perfect time to extubate is important to prevent MV complications [2]. There are multiple predictors that are used to predict the perfect timing of MV removal [6]. One of the most common predictors, which was first described by Yang and Tobin, is the rapid shallow breathing index (RSBI) [7]. RSBI for adult group is defined as the ratio of respiratory rate (RR) over tidal volume (VT) [8]. For pediatric group, same equation for RSBI is used with a correction by weight, and it is usually calculated during a two-hour spontaneous breathing trial (SBT) [9]. Multiple studies considered RSBI lower than 105 breath/min/L as a predictor for successful extubation in adult group [10-12]. Although RSBI threshold has been calculated and defined for adults, the threshold among pediatric patients still unclear yet [5]. Also, several studies suggested that multiple measurements of the RSBI may be more predictive than single measurement [6,13]. Thus, our aim in this study is to evaluate the RSBI as a predictor of extubation outcome in pediatric patients underwent cardiac surgery at King Faisal Cardiac Center from 2016 until 2019.

## Materials and methods

This is a retrospective cohort study that included all pediatric patients within the age of birth till 14 years who underwent extubation post cardiac surgeries from 2016 to 2019. The study took place at King Faisal Cardiac Center. Patients admitted for reasons other than cardiac surgeries were excluded. Data was gathered using a data collection sheet used to collect RSBI and other variables like (demographics, vital signs, respiratory parameters, pre-extubation ventilator settings, complete blood count, and surgical complications). All data was acquired from Best Care System using electronic medical records at King Faisal Cardiac Center. Three readings of RSBI on hourly basis prior to extubation were calculated by dividing RR over VT with correction based on the body weight (RR/VT/Kg). Calculations were done two hours before extubation, one hour before extubation, and at the time of extubation. All of the three RSBI measurements were calculated during the two-hours SBT in which the patients were receiving no ventilatory support. Variables other than RSBI were collected in the last hour prior to extubation. Patients were categorized into three outcome groups. The three groups include the extubation success group, group of patients who succeeded extubation with the need of non-invasive ventilation, and the extubation failure group. Extubation failure was defined as the need of reintubation within 48 hours after extubation.

The sample size was calculated for two independent groups. Incidence rate of the extubation failure group was 12% [14]. The anticipated extubation success group has 22% incidence rate with difference of 10%. Alpha value is 0.05, and the power of the study was 80%. The required minimum sample size was determined to be 220 patients for each group. The total number was 440 patients. However, a non-probability consecutive sampling technique was used to include all patients who met the inclusion and exclusion criteria from 2016 to 2019. The data was collected by co-authors of the study. Ethical approval was taken from institutional review board, and scientific approval was taken from King Abdullah International Medical Research Center.

IBM SPSS version 23 (IBM Corp., Armonk, NY) was used for data analysis. Categorical variables were reported as percentages, and numerical variables were reported as means or medians. Normally distributed numerical variables were reported using the mean and standard deviation (SD), while abnormally distributed variables were reported using the median and interquartile range (IQR). Kruskal-Wallis test was used to analyze the relation between the three outcome groups and the three readings of RSBI measurements by comparing the means. Other pre-extubation ventilator settings and vital signs were also compared to the outcome by using either Anova test or Kruskal-Wallis test depending on the normality of distribution. P-value lower than 0.05 was considered significant.

## Results

**Figure 1:**
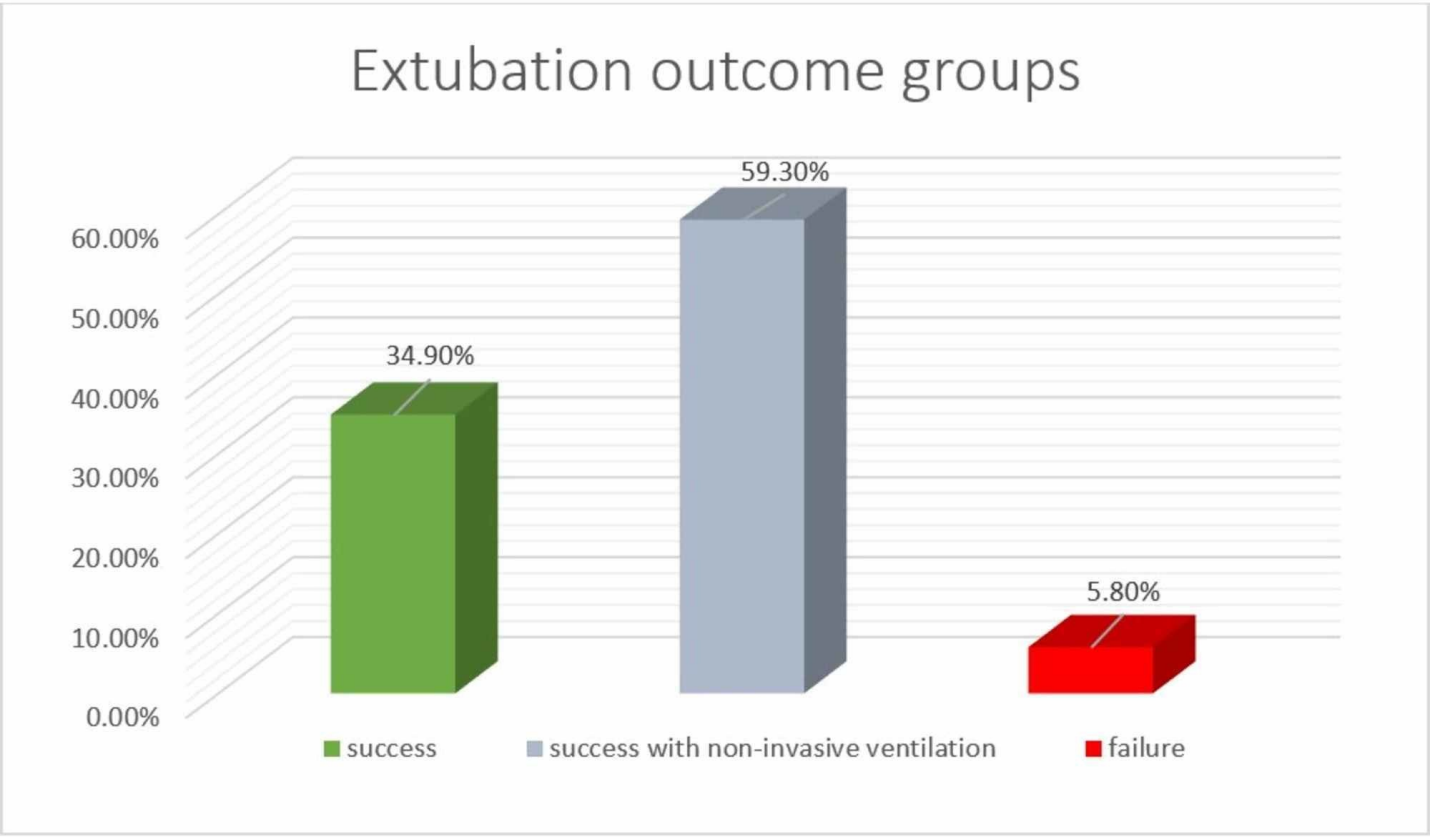
Ratio of the extubation outcome groups of pediatric patients underwent cardiac surgeries.

In this retrospective cohort study, 86 patients met the inclusion and exclusion criteria. Median age was six months with the same interquartile range (IQR) as six months. Median weight was 4.9 kg with IQR 3.21 kg. In terms of gender, 40 patients (46.5%) were males and 46 patients (53.5%) were females. For the outcome groups, 30 patients were successfully extubated with a ratio of 34.9%, 51 patients had successful extubation with the use of non-invasive ventilation and had a ratio of 59.3%, and only five patients suffered from extubation failure with a ratio of 5.8% (Figure 1).

Post-surgical complications and risk factors were observed in the outcome groups variably as shown in Table 1.

**Table 1:** Post-surgical complications and risk factors associated with the study population.

Surgical complications and risk factors	Extubation outcome groups			
	Success	Success with non-invasive ventilation	Failure	Total
Infection	6	5	1	12
Residual cardiac lesion	0	0	1	1
Pleural effusion	0	0	1	1
Thrombosis	0	0	1	1
Heart block	2	3	0	5
Diaphragmatic paralysis	0	4	0	4

All of the three RSBI that were measured in different periods were abnormally distributed. The median and IQR for the three measurements is shown in Table 2.

**Table 2:** Median and IQR for the three RSBI measurements. RSBI: Rabid shallow breathing index; IQR: Interquartile range.

RSBI period of time	Median	IQR
2 hours before extubation	4	3.79
1 hour before extubation	4.14	3.60
At time of extubation	3.82	3.49

Regarding the three RBSI measurements as predictors and their association with the outcome groups, two hours before extubation had a P-value of 0.003, RSBI one hour before extubation had a P-value of 0.01, and RSBI at time of extubation had a P-value of 0.02. The exact mean rank of the three RSBI measurements for every outcome groups is demonstrated in Table 3.

**Table 3:** RSBI as predictor for the extubation outcome of pediatric patients underwent cardiac surgery. RSBI: Rabid shallow breathing index; n: number of patients. Kruskal-Wallis test was used to find the statistical significance between variables.

Outcomes		n	Mean rank	P-value
RSBI (breath/min/ml/Kg) 2 hours before extubation	Success	30	31.23	0.003
	Success with non-invasive ventilation	51	49.57	
	Failure	5	55.20	
	Total	86		
RSBI (breath/min/ml/Kg) 1 hour before extubation	Success	30	32.50	0.01
	Success with non-invasive ventilation	51	49.98	
	Failure	5	43.40	
	Total	86		
RSBI (breath/min/ml/Kg) at time of extubation	Success	30	33.23	0.02
	Success with non-invasive ventilation	51	49.16	
	Failure	5	47.40	
	Total	86		

Different modes of MV were used before extubation. For the extubation failure group, pressure support ventilation was used in four of the five failure cases, and synchronized intermittent mechanical ventilation was used in one of the five failure cases. Other outcome groups and modes of MV used are shown in Table 4.

**Table 4:** Modes of mechanical ventilation (MV) before extubation for outcome groups. PSV: Pressure support ventilation; PRVC: Pressure-regulated volume control; SIMV: Synchronized intermittent mechanical ventilation; CPAP-PS: Continuous positive airway pressure-pressure support.

Outcomes	Modes of MV before extubation				
	PSV	Pressure control + volume guarantee	PRVC	SIMV	Spontaneous breathing CPAP-PS
Success	17	2	1	7	3
Success with non-invasive ventilation	32	0	0	11	8
Failure	4	0	0	1	0
Total	53	2	1	19	11

The mean and standard deviation (SD) of normally distributed pre-extubation ventilator settings, vital signs, and Risk Adjustment in Congenital Heart Surgery (RACHS) score for each outcome group are shown in Table 5.

**Table 5:** Normally distributed pre-extubation ventilators settings, vital signs, RACHS score and its association with the outcome groups. SD: Standard deviation; n: number of patients; HR: Heart rate; BP: Blood pressure; FiO2: Fraction of inspired oxygen; PaCO2: Partial pressure of carbon dioxide; O2 Sat: Oxygen saturation; Hb: Hemoglobin; RACHS: Risk adjustment in congenital heart surgery. Anova test was used to find the statistical significance between variables.

	Outcome group	n	Mean ± SD	p-value
HR (bpm)	Success	30	125.53 ± 21.18	0.136
	Success with non-invasive ventilation	51	120.06 ± 24.20	
	Failure	5	140.40 ± 17.67	
	Total	86	123.15 ± 23.19	
BP (mmHg)	Success	30	93.70 ± 12.60	0.060
	Success with non-invasive ventilation	51	87.16 ± 13.16	
	Failure	5	84.20 ± 7.29	
	Total	86	89.27 ± 13.02	
FiO2	Success	30	28.80 ± 5.66	0.173
	Success with non-invasive ventilation	51	31.80 ± 7.76	
	Failure	5	30.00 ± 3.53	
	Total	86	30.65 ± 7	
PaCO2 (mmHg)	Success	29	42.48 ± 7.15	0.462
	Success with non-invasive ventilation	50	44.84 ± 9.06	
	Failure	5	45.40 ± 9.20	
	Total	84	44.06 ± 8.43	
O2 sat (%)	Success	30	95.53 ± 5.34	0.134
	Success with non-invasive ventilation	50	91.99 ± 8.75	
	Failure	5	93.56 ± 5.41	
	Total	85	93.33 ± 7.66	
Hb (g/dl)	Success	30	10.753 ± 1.52	0.392
	Success with non-invasive ventilation	51	10.927 ± 1.36	
	Failure	5	10.040 ± 1.01	
	Total	86	10.815 ± 1.40	
RACHS score	Success	30	2.40 ± 0.56	0.295
	Success with non-invasive ventilation	50	2.54 ± 0.90	
	Failure	5	3.00 ± 0.70	
	Total	85	2.52 ± 0.79	

Other abnormally distributed pre-extubation settings, and the inotropic score and the association with outcome groups are shown in Table 6.

**Table 6:** Abnormally distributed pre-extubation settings, and the inotropic score. n: number of patients; PaO2: Partial pressure of oxygen. Kruskal-Wallis test was used to find the statistical significance between variables.

Outcomes		n	Mean rank	P-value
PaO2 (mmHg)	Success	29	49.34	0.167
	Success with non-invasive ventilation	50	38.58	
	Failure	5	42	
	Total	84		
Inotrope score	Success	30	43.10	0.990
	Success with non-invasive ventilation	51	43.63	
	Failure	5	44.60	
	Total	86		
Oxygen index	Success	29	36.81	0.276
	Success with non-invasive ventilation	50	45.08	
	Failure	5	49.70	
	Total	84		

Mean corpuscular volume (MCV) was statistically significant compared to the outcome groups (P-value = 0.01). MCV mean ± SD for success group is 82.57 ± 4.6, 84.86 ± 4.48 for success with non-invasive ventilation group, and 88.20 ± 7.22 for the failure group as shown in Table 7.

**Table 7:** Mean corpuscular volume (MCV) as a predictor of extubation outcome of pediatric patients underwent a cardiac surgery. SD: Standard deviation; n: number of patients. Anova test was used to find the statistical significance between variables.

Outcomes	n	Mean ± SD	P-value
Success	30	82.57 ± 4.61	0.01
Success with non-invasive ventilation	51	84.86 ± 4.48	
Failure	5	88.20 ± 7.22	
Total	86	84.26 ± 4.86	

## Discussion

Although MV is essential in critical care patients to maintain lives, extubation failure and the need of reintubation has always been a concern due to the devastating complications such as pneumonia, prolong hospital stay, and increase in mortality [15,16]. To date, there is no definite protocol to establish the weaning of MV in pediatric patients after surgeries. In mechanically ventilated pediatric patients in general, the ratio of extubation failure is obviously variable in different studies ranging from 4.1% to 14% [17-19]. The ratio of extubation failure for pediatric patients underwent cardiac surgery at King Faisal Cardiac Center from 2016 to 2019 is 5.8%, which is in between the various ratios that have been reported in literature. However, in comparing the ratio in this study with other two studies that focused only on pediatric patients underwent cardiac surgeries, our extubation failure ratio is considered lower than both of the studies [20,21]. One of these studies about pediatric cardiac patients had an extubation failure ratio of 19% [20].

In our study, for the extubation success group, most of them were requiring the use of non-invasive ventilation after the removal of MV, and they represent 63% of patients who did not suffer from extubation failure. The association between extubation outcomes and the earliest RSBI measurement which was measured two hours prior to extubation had the lowest and the most statistically significant p-value in comparison to the two other RSBI measurements. In the RSBI measurement that was calculated before two hours of extubation, the low values of RSBI were more associated with the success group with an RSBI mean rank of 31.23 breath/min/ml/kg, and the failure group had the highest RSBI with a mean rank of 55.20 breath/min/ml/kg. The group who had a successful extubation with the use of non-invasive ventilation had an RSBI mean rank of 49.75 breath/min/ml/kg which is in the middle between the success and the failure group. For the other two RSBI measurements, the one-hour prior to extubation and at-extubation RSBI measurements, the success group also had the lowest RSBI measurements. Although the association between extubation outcome and the latter two RSBI measurements prior to extubation was statistically significant, the success with non-invasive ventilation in these two periods had a mean rank of RSBI slightly higher than the failure group which is not similar to the results in the earliest RSBI measurements, the two hours prior to extubation. However, these high RSBI in the last two measurements could be considered as an alert to start the use of non-invasive ventilation, and helping the patient to succeed the extubation, and this was not done for the failure group whom had slightly lower RSBI than the success with non-invasive ventilation group.

Similar to our study, a study was done in Portugal specifically for pediatric patients underwent cardiac surgery about evaluating multiple predictors used to predict extubation outcome. This similar study revealed no association between the RSBI measurements and extubation outcomes [20]. In other studies about pediatric patients in general, they found an association between the measurements of RSBI prior to extubation and the extubation outcomes which is a prediction of extubation success with low RSBI similar to the results in the two hours prior to extubation RSBI measurements in our study [9,10,22,23]. Unlike our study, some of these studies established a threshold, cut-off point, to predict the successful extubation, but these cut-off points were variable and ranging from 6.7 to 11 [9,10,23].

Other variables that were measured an hour before extubation which includes heart rate, blood pressure, fraction of inspired oxygen, partial CO2 and O2, oxygen index, oxygen saturation, and hemoglobin revealed no difference between the outcome groups with no statistical significance and could not be used as a predictor for extubation success or failure. However, an association was found between the mean corpuscular volume (MCV) and the outcome groups. The extubation failure group had higher MCV mean than the extubation success group with extremely significant p-value. Up to our knowledge, no studies about the use of MCV as a predictor of extubation outcomes was done before. Although we found a statistical association between the extubation failure and the low level of MCV, it is still not clear in clinical setting.

## Conclusions

MV is widely used after pediatric cardiac surgeries and, some of those patients may be reintubated after extubation. A reliable tool or measurements to predict the correct timing to extubate the pediatric patients is still not yet discovered. Our study suggests that pediatric patients who suffer extubation failure usually have a higher RSBI measurement compared to the patients who have a successful extubation. The most significant RSBI measurements to predict the extubation outcome were recorded earlier two hours prior to extubation. The use of non-invasive ventilation after extubation could be a tool to prevent the failure for high measurement. Our study also found that extubation failure patients could have MCV higher than the success group although it is clinically not significant. A cut-off RSBI measurement to predict the extubation outcome was not identified due to the small sample size. A similar prospective future study can be done to be more specific about the relation of RSBI and extubation outcomes.
